# Treatment compliance and effectiveness in complex PTSD patients with co-morbid personality disorder undergoing stabilizing cognitive behavioral group treatment: a preliminary study

**DOI:** 10.3402/ejpt.v4i0.21171

**Published:** 2013-11-06

**Authors:** Ethy Dorrepaal, Kathleen Thomaes, Johannes H. Smit, Dick J. Veltman, Adriaan W. Hoogendoorn, Anton J. L. M. van Balkom, Nel Draijer

**Affiliations:** 1 GGZ inGeest, Amsterdam, The Netherlands; 2 Department of Psychiatry, VU University Medical Center, Amsterdam, The Netherlands; 3 EMGO Institute, VU University Medical Center, Amsterdam, The Netherlands; 4 PsyQ, Parnassiagroep, The Hague, The Netherlands

**Keywords:** Personality disorders, treatment outcome, child abuse, posttraumatic stress disorder, cognitive behavioral treatment, complex PTSD, dissociative subtype

## Abstract

**Background:**

In the empirical and clinical literature, complex posttraumatic stress disorder (PTSD) and personality disorders (PDs) are suggested to be predictive of drop-out or reduced treatment effectiveness in trauma-focused PTSD treatment.

**Objective:**

In this study, we aimed to investigate if personality characteristics would predict treatment compliance and effectiveness in stabilizing complex PTSD treatment.

**Method:**

In a randomized controlled trial on a 20-week stabilizing group cognitive behavioral treatment (CBT) for child-abuse-related complex PTSD, we included 71 patients of whom 38 were randomized to a psycho-educational and cognitive behavioral stabilizing group treatment. We compared the patients with few PD symptoms (adaptive) (*N*=14) with the non-adaptive patients (*N*=24) as revealed by a cluster analysis.

**Results:**

We found that non-adaptive patients compared to the adaptive patients showed very low drop-out rates. Both non-adaptive patients, classified with highly different personality profiles “withdrawn” and “aggressive,” were equally compliant. With regard to symptom reduction, we found no significant differences between subtypes. *Post-hoc*, patients with a PD showed lower drop-out rates and higher effect sizes in terms of complex PTSD severity, especially on domains that affect regulation and interpersonal problems.

**Conclusions:**

Contrary to our expectations, these preliminary findings indicate that this treatment is well tolerated by patients with a variety of personality pathology. Larger sample sizes are needed to study effectiveness for subgroups of complex PTSD patients.

Child physical abuse and rape are known to result in a very high prevalence (50%) of posttraumatic stress disorder (PTSD) (Kessler, Sonnega, Bromet, Hughes, & Nelson, 1995), frequently complicated by additional sequelae-like problems in affect regulation, memory and attention, self-perception, interpersonal relations, somatization and systems of meaning (Herman, 1992), referred to as “PTSD with associated features” in DSM-IV-TR or “complex PTSD” (Pelcovitz et al., 1997). Empirical studies as well as neurobiological findings have supported the distinction of complex PTSD from “simple” PTSD (Thomaes et al., 2010, 2013; Van Der Kolk, Roth, Pelcovitz, Sunday, & Spinazzola, 2005).

Standard trauma-focused cognitive behavioral treatment has proven to be effective for PTSD (Bradley, Greene, Russ, Dutra, & Westen, 2005; Foa, Keane, Friedman, & Cohen, 2009), although drop-out rates in these studies are high (approximately 30%). In contrast, the effectiveness of treatments for child-abuse-related complex PTSD or PTSD with personality disorder (PD), has not been well studied (Bradley et al., 2005; Cloitre, 2009). Some data suggest that immediate trauma-focused treatment may be less efficacious and less well tolerated in these populations (Ehlers et al., 1998; Ford & Kidd, 1998; McDonagh et al., 2005; Tarrier et al., 1999; Van Der Kolk et al., 2007).

There is a clinical need for suitable treatments for patients with child-abuse-related complex PTSD. Stabilizing treatment addresses additional sequelae-like affect-dysregulation before the start of trauma focused CBT (Chard, 2005; Cloitre et al., 2010; Dorrepaal et al., 2012b; Zlotnick et al., 1997). Such a phase-based approach as advocated by experts (Cloitre et al., 2011) aims at integrating the potentially complementary advantages of exposure-based methods with the benefits of affect-management models while limiting potential drawbacks of both treatments separately.

However, although tailoring treatment may thus be clinically relevant, child-abuse-related complex PTSD shows high inter-subject variability in psychiatric co-morbidity. Some patients have internalizing personality traits and may present themselves as inhibited, anxious and withdrawn, whereas others with externalizing personality characteristics appear disinhibited, angry, and affect-dysregulated (Allen, Huntoon, & Evans, 1999; Miller & Resick, 2007). Such co-morbid personality disturbances may explain differences in treatment results (Allen, Coyne, & Huntoon, 1998; Hyer, Davis, Albrecht, Boudewyns, & Woods, 1994; Talbot, Duberstein, Butzel, Cox, & Giles, 2003).

In a previous study (Dorrepaal et al., 2012a) we reported a cluster analysis of PD symptoms, which resulted in five complex PTSD subtypes. First, we identified a relatively “adaptive” subtype associated with the fewest PD symptoms. The non-adaptive subgroups differed on dimensions of “introversion” and “disinhibition” resulting in four non-adaptive subgroups. Of these, the “withdrawn” and “aggressive” subtypes showed the most qualitatively distinct patterns, prototypical for “internalizers” and “externalizers,” respectively.

In this study, we aimed to investigate whether these subtypes differed with respect to (1) compliance as well as (2) improvement when treated with a 20-week stabilizing group cognitive behavioral treatment (CBT). We expected that the “adaptive” patients would show the lowest drop-out rate and that the “non-adaptive” patients in the “aggressive” subgroup would show the highest drop-out rate, since all these patients met the criteria for a co-morbid borderline PD. Furthermore, we expected that the “withdrawn” patients, who were most self-defeating, would show poor treatment outcome.

## Method

### Study design randomized controlled trial

In a multi-site, randomized controlled trial (RCT) in which a total of 71 outpatients were enrolled, two treatment conditions were compared: (1) psycho-educational and cognitive behavioral stabilizing group treatment added to treatment as usual (TAU; *N*=38) and (2) TAU only (*N*=31) (Dorrepaal et al., 2012b). For this study, we used the data of the 38 patients with child-abuse-related complex PTSD who were treated with stabilizing group treatment across four sites.

#### Intervention

The treatment protocol consisted of 20 weekly 2-hour meetings in addition to individual TAU (Dorrepaal, Thomaes, & Draijer, 2008). The groups consisted of 8–12 participants and two therapists. During the first 10 sessions, psycho-education was provided, as well as registration homework to recognize “triggers.” Psycho-education aimed at attaining a sense of cognitive mastery by explaining symptoms as adaptations once necessary to emotional survival in a context of child abuse. Subsequently, intentional avoidance to acquire greater cognitive and emotional control was taught, followed by skills training to learn to tolerate negative feelings and ultimately decrease avoidance. Sessions 11–20 contained cognitive restructuring, on topics such as problems with anger, assertiveness, bodily experiences, shame, guilt, and distrust. The group format aimed at inducing hope and reframing patients’ symptoms as normal responses to trauma, thereby reducing shame, guilt, and isolation. Taken together, increased self-regulation, more adaptive beliefs, and improved interpersonal functioning to enhance stability were goals of the treatment.

The treatment manual consisted of a highly structured, detailed session-by-session script (psycho-educational presentations on a weekly topic, related skills training, assignments, and homework pages). The material in the stabilizing group treatment consisted of a tested manual by Zlotnick et al., (1997); Wolfsdorf & Zlotnick, 2001; Zlotnick et al., 1995). Three of the authors (Dorrepaal et al., 2008) extended this protocol by inserting additional sessions (14–19) to cover all symptom domains of complex PTSD.

### Inclusion criteria

Female patients who experienced sexual and/or physical abuse before the age of 16 who met the criteria for both PTSD (DSM-IV) and complex PTSD (assessed with the Structured Interview of Disorders of Extreme Stress; SIDES, Pelcovitz et al., 1997) were enrolled in the study. Exclusion criteria included the presence of antisocial PD, current psychotic episode, dissociative identity disorder or severe alcohol or drug dependence or abuse (likely to interfere with attendance) as assessed by Structured Clinical Interviews for DSM: SCID-I (First et al., 1986) and SCID-D amnesia part (Steinberg, Rounsaville, & Cicchetti, 1990). Subjects currently under exposure treatment or seeking such treatment were also excluded. The Davidson Trauma Scale (DTS; Davidson, Tharwani, & Connor, 2002) was used to measure PTSD severity. The SIDES was used to assess the severity of complex PTSD. Presence and severity of PDs were assessed with the Structured Interview for DSM-IV Axis II Personality disorders (SIDP-IV) (Pfohl, Blum, & Zimmerman, 1997).

### Outcome RCT

After 20 weeks, both treatments were associated with significant improvement. However, the pre-to-post effect size for the experimental group treatment was large and that for the control condition was medium. Analyses of completers data found that *t*-tests of change scores were greater for the experimental group treatment than for the control condition in regard to the SIDES (*p*=0.05) with a similar trend for the DTS (*p*=0.10). Similarly, categorical analyses among completers revealed more responders in the experimental group on both the DTS (55% vs. 24%; *p*= 0.02) and SIDES (74% vs. 50%; *p*= 0.05).

Analyses of the intent to treat sample found that *t*-tests of change scores did not reach statistical significance. However, in the intention to treat analysis, there were significantly more responders in the experimental group on the DTS (45% vs. 21%; *p*=0.03), but not on SIDES. Overall, responder analyses indicate the clinical meaningfulness of adding group treatment (Dorrepaal et al., 2012b).

### Subjects

The 38 patients included at the four treatment sites of this study showed severe PTSD symptoms at pre-test (DTS mean: 90, SD: 20). Most patients (76%) suffered one or more Axis II diagnosis (mainly borderline, 53%; avoidant, 21%, obsessive compulsive, 13%, and the proposed depressive PD, 18%).

In a previous study, we applied Ward's hierarchical cluster-analysis on DSM-IV Axis II features using the SIDP-IV severity scores (Dorrepaal et al., 2012a), first yielding two clusters: adaptive versus non-adaptive. Second, the non-adaptive cluster was split in four sub clusters: withdrawn, alienated, suffering, and aggressive. The 38 patients were distributed as follows: adaptive (*n*=14; 37%), non-adaptive (*n*=24; 63%), withdrawn (*n*=4; 17%); alienated (*n*=1; 5%); suffering (*n*=9; 37%); and aggressive (*n*=10; 41%).

### Adaptive vs. non-adaptive subtypes

The adaptive and the non-adaptive subtypes differed with respect to borderline, avoidant, self-defeating and depressive features, as well as Cluster A, B, and C personality pathology. Almost half of the patients belonging to the adaptive subtype met the criteria for a PD versus almost all patients belonging to the non-adaptive subtype. Additionally, the adaptive patients had lower DES scores (mean 18 as compared to mean 30 in non-adaptive patients).

### Non-adaptive subtypes

The non-adaptive subtypes were split on an “introversion” dimension (PDs with avoidant and dependent symptoms) and a “disinhibition” dimension (PDs with borderline and antisocial symptoms). The four non-adaptive subtypes were characterized as follows:

1. Withdrawn subtype: introvert and not disinhibited. This subtype was characterized by high levels of avoidance, dependence, self-defeating, and depressive personality symptoms.
2. Alienated subtype: both introvert and disinhibited. This subtype showed very high ratings across all DSM-IV Clusters. A uniquely high score was found on Cluster A PDs (schizotypal, schizoid, paranoid), indicating introversion and paranoia, combined with very high levels of borderline features, indicating disinhibition.
3. Suffering subtype: not introvert and not disinhibited. This subtype showed intermediate levels on both DSM-IV Cluster B and C symptoms relative to the most disturbed (alienated) subtype, on the one hand, and the adaptive subtype, on the other hand.
4. Aggressive subtype: not introvert and disinhibited. High scores on DSM-IV Cluster B borderline symptoms accompanied by low Cluster C avoidance and dependence symptoms.

### Data analyses

1. To examine whether subtypes predicted drop-out, logistic regression was used with adaptive subtype (yes/no) as independent variable and drop-out rate as dependent variable.
2. Adaptive versus non-adaptive subtypes were evaluated on treatment effect in terms of PTSD and complex PTSD symptoms using linear regression analysis with change scores (pre-test minus post-test) as the dependent variable among the completers. Cohen's *d* effect sizes (ES) were computed. Medium ES > 0.5 were considered meaningful and are reported. All computations were carried out with SPSS 15.0.

## Results

### Does personality-based subtype predict drop-out?

Of the 38 patients randomized to a psycho-educational and cognitive behavioral stabilizing group treatment, seven (18%) dropped out prematurely, leaving 31 completers. Drop-out versus completer analysis revealed no significant differences on pre-test variables. Contrary to our hypothesis, the adaptive subtype showed by far the highest drop-out percentage (43%), as opposed to the non-adaptive clusters in which drop-out was very low (4%) (logistic regression: β = 2.84, SE β = 1.15, *p =*0.01, *R* ^2^=0.208 (Cox & Snell); 0.338 (Nagelkerke), Chi-square 8.9, *df*=1, *p =*0.003, two-tailed). *Post-hoc* no significant differences between the drop-out rates in the prototypical subtypes “withdrawn” (*N*=4; 0%) versus “aggressive” (*N*=10; 10%) were observed (likelihood ratio, 0.70; *df*=1, *p =*0.40).

### Does personality-based subtype predict treatment effect?

Overall Cohen's *d* (ES) on the PTSD severity was large (ES = 1.1). The mean change score on the DTS was 24.7 (SD 27.3). No significant differences between adaptive versus non-adaptive subtypes in change scores were found (linear regression: β = 1.53, SE β = 5.68, *t =*0.27, *p =*0.79). Change scores varied from 13.8 (SD 31.1) in the aggressive cluster to 29.4 (SD 23) in the suffering cluster (with a non-significant difference between these two clusters with an ES of 0.6).

Moreover, Cohen's *d* ES on the SIDES (complex PTSD severity) was large as well (ES = 1.8). The SIDES mean change score was 14.7 (SD 10.6). Again, no significant differences between the adaptive subtypes versus the non-adaptive subtypes were found (β = 1.63, SE β = 2.18, *t =*0.74, *p =*0.46). Change scores varied from 11.6 (SD 8.8) in the aggressive cluster to 17.1 (SD 9.2) in the adaptive cluster (with a non-significant difference between these clusters with an ES of 0.7). Based on ES > 0.5, improvement was most evident on SIDES subscales self-esteem and interpersonal problems for the withdrawn subtype, and on affect regulation for the adaptive and suffering subtype.

Contrasting the prototypical subtypes “withdrawn” versus “aggressive” did not reveal a significant difference on change scores of DTS or SIDES, and ES were smaller than 0.5 (data not shown).

### *Post-hoc* analyses on Axis II co-morbidity

#### Is the presence of co-morbid Axis II diagnosis related to drop-out?

To further investigate the puzzling finding that on the one hand, a large percentage (43%) of the adaptive subgroup dropped out of treatment, while on the other hand the adaptive patients who completed treatment obtained favorable treatment results, a comparison was carried out between completers and drop-outs within the adaptive cluster. We found that the adaptive drop-outs showed a 33% prevalence of co-morbid Axis II diagnosis compared with 75% in the adaptive completers (likelihood ratio, 2.49; *df*=1; *p =*0.12).

Across subtypes, the absence of Axis II diagnosis was associated with 44% drop-out versus 10% drop-out when co-morbid Axis II diagnoses were present (Chi-square: 5.3, *df*=1, *p =*0.02). A similar pattern was found for patients with borderline PD as well as avoidant PD.

#### Does the presence of co-morbid Axis II diagnosis predict treatment effect?

PTSD change scores did not differ significantly between patients with versus those without a diagnosis of a PD: *t =*0.13, *df*=29, *p =*0.90). In addition, the between-group ES was smaller than 0.5.

With respect to change scores on complex PTSD symptoms, patients with Axis II diagnoses showed a mean change score of 15.5 (SD 11.1), compared to a mean change score of 10.5 (SD 5.4) in patients without Axis II diagnoses. This difference was statistically not significant (*t =*−0.98, *df*=29, *p =*0.33). The between-group ES Cohen's *d* in patients with Axis II diagnoses was 0.5 higher compared with patients without Axis II diagnoses.

## Discussion

In this study, we aimed to investigate if personality-based subtypes within a complex PTSD population would predict differential treatment compliance and effectiveness. Contrary to our expectations, we found that non-adaptive patients with the most severe personality pathology showed the lowest drop-out rates (4%) during stabilizing group CBT.. In contrast, the adaptive patients were found to drop out frequently (43%). In addition, two most contrasting types of non-adaptive patients – withdrawn and aggressive – were found to be equally compliant, suggesting that this treatment is well tolerated by patients with a variety of personality pathology. With regard to PTSD as well as complex PTSD symptom reduction, however, we found no significant differences between adaptive and non-adaptive subtypes, all achieving good results, or between various subtypes of non-adaptive patients, although the latter result may have been due to low power.

*Post-hoc* analyses showed that co-morbid Axis II diagnoses were related to *enhanced* treatment compliance. Our drop-out rate in patients with co-morbid PD (10%) was low compared to drop-out rates in specific PD treatments, ranging from 25 to 51% (Doering et al., 2010). Moreover, our patients with PDs showed larger effect sizes in terms of complex PTSD symptoms compared to patients without PD.

To our knowledge, this is the first study reporting enhanced PTSD treatment compliance in a specific complex PTSD population: non-adaptive patients with co-morbid PD. Additionally, besides more severe personality pathology, the level of dissociation was higher in the non-adaptive groups as well (Dorrepaal et al., 2012a). This might indicate overlap with the newly appeared DSM-5 dissociative subtype, also shown in neurobiological studies (e.g., Thomaes et al., 2010, 2013) and suggest its predictive value. Note however, that these results should be considered highly preliminary due to the small sample size. Replication of our study in a larger sample would be very useful in order to examine whether the results obtained remain stable with larger groups.

Compliance may be associated with type of treatment. Factors that may be important include first the integrated approach in which not only PTSD symptomatology but also associated pathology was a focus of the treatment. Second, the group format is presumably an important feature, and it is likely to be effective in relieving stigma, isolation, and shame, while improving social relationships and self-esteem by recognition and learning from each other. Third, the primary focus of the treatment was on stabilization by psycho-education and increasing affect regulation skills, only later followed by cognitive restructuring on directly trauma-related and interpersonal issues, which may have attributed to enhanced compliance. A comparable paced treatment schedule, starting with affect-management followed by narration and cognitive reappraisal of trauma memories has likewise been shown to be effective (Cloitre et al., 2010). These treatments can be considered as examples of treatment extensions for patients with co-morbid PDs beyond the standard manual for Axis I disorders (Weertman, Arntz, Schouten, & Dreessen, 2005). In contrast, for many adaptive patients without co-morbid PD, the content, duration, (group) format or heterogeneous elements of stabilizing treatment may not provide a good match, thereby resulting in high drop-out rates, and other treatments may suit these patients better. Note, however, that adaptive completers reached good results indicating that (part of) this treatment may be valuable for them as well.

These results contrast sharply with previous findings on trauma-focused treatment, showing that the presence of Axis II *disorders* – especially BPD – was associated with enhanced drop-out rates (McDonagh et al., 2005), while studies investigating borderline *features* (Clarke, Rizvi, & Resick, 2008; Feeny, Zoellner, & Foa, 2002; Karatzias et al., 2007; Van Minnen, Arntz, & Keijsers, 2002) in trauma-focused treatment have found no differential drop-out rates or treatment effects, but this latter finding probably does not generalize to the more severe BP *Disorder* patients. Taken together, these findings indicate that trauma-focused treatment is most suitable and effective for PTSD patients without co-morbid PD, whereas stabilizing CBT is appropriate for non-adaptive child-abused patients with co-morbid PD. Axis II co-morbidity may therefore be considered a “prescriptive” variable that predicts a different pattern of treatment outcome between various treatment modalities (Olatunji, Cisler, & Deacon, 2010; Resick et al., 2008). Future research aimed at a direct comparison of initially stabilizing versus immediate trauma-focused treatment in complex PTSD patients with co-morbid PD in terms of compliance as well as treatment effectiveness is warranted to corroborate these preliminary findings.

In conclusion, these preliminary findings may aid in providing optimal treatment to patients with child-abuse-related (complex) PTSD. When Axis II disorders are absent, drop-out risk is high in a 20-week stabilizing group CBT, whereas in patients with co-morbid Axis II diagnosis, drop-out risk is likely to be low compared to trauma-focused cognitive behavioral treatment. Such considerations would be in line with the rationale of a phased approach in Complex PTSD patients: stabilization if necessary; trauma focused if possible.

## References

[CIT0001] Allen J. G, Coyne L, Huntoon J (1998). Complex posttraumatic stress disorder in women from a psychometric perspective. Journal of Personality Assessment.

[CIT0002] Allen J. G, Huntoon J, Evans R. B (1999). Complexities in complex posttraumatic stress disorder in inpatient women: Evidence from cluster analysis of MCMI-III Personality Disorder Scales. Journal of Personality Assessment.

[CIT0003] Bradley R, Greene J, Russ E, Dutra L, Westen D (2005). A multidimensional meta-analysis of psychotherapy for PTSD. American Journal of Psychiatry.

[CIT0004] Chard K. M (2005). An evaluation of cognitive processing therapy for the treatment of posttraumatic stress disorder related to childhood sexual abuse. Journal of Consulting and Clinical Psychology.

[CIT0005] Clarke S. B, Rizvi S. L, Resick P. A (2008). Borderline personality characteristics and treatment outcome in cognitive-behavioral treatments for PTSD in female rape victims. Behavior Therapy.

[CIT0006] Cloitre M (2009). Effective psychotherapies for posttraumatic stress disorder: A review and critique. CNS Spectrums.

[CIT0007] Cloitre M, Courtois C. A, Charuvastra A, Carapezza R, Stolbach B. C, Green B. L (2011). Treatment of complex PTSD: Results of the ISTSS expert clinician survey on best practices. Journal of Traumatic Stress.

[CIT0008] Cloitre M, Stovall-McClough K. C, Nooner K, Zorbas P, Cherry S, Jackson C. L (2010). Treatment for PTSD related to childhood abuse. A randomized controlled trial. American Journal of Psychiatry.

[CIT0009] Davidson J. R, Tharwani H. M, Connor K. M (2002). Davidson Trauma Scale (DTS): Normative scores in the general population and effect sizes in placebo-controlled SSRI trials. Depression and Anxiety.

[CIT0010] Doering S, Horz S, Rentrop M, Fischer-Kern M, Schuster P, Benecke C (2010). Transference-focused psychotherapy v. treatment by community psychotherapists for borderline personality disorder: Randomised controlled trial. British Journal of Psychiatry.

[CIT0011] Dorrepaal E, Thomaes K, Draijer N (2008). Vroeger en verder, stabilisatiecursus na misbruik of mishandeling. Handleiding.

[CIT0012] Dorrepaal E, Thomaes K, Smit J. H, Hoogendoorn A. W, Veltman D. J, Draijer N (2012a). Clinical phenomenology of childhood abuse-related complex PTSD in a population of female patients: Patterns of personality disturbance. Journal of Trauma and Dissociation.

[CIT0013] Dorrepaal E, Thomaes K, Smit J. H, Van Balkom A. J. L. M, Veltman D. J, Draijer N (2012b). Stabilizing group treatment for complex posttraumatic stress disorder related to child abuse: A randomized controlled trial. Journal of Psychosomatics and Psychotherapy.

[CIT0014] Ehlers A, Clark D. M, Dunmore E, Jaycox L, Meadows E, Foa E. B (1998). Predicting response to exposure treatment in PTSD: The role of mental defeat and alienation. Journal of Traumatic Stress.

[CIT0015] Feeny N. C, Zoellner L. A, Foa E. B (2002). Treatment outcome for chronic PTSD among female assault victims with borderline personality characteristics: A preliminary examination. Journal of Personality Disorders.

[CIT0016] First M. B, Spitzer R. L, Williams J. B. W, Gibbon M (1986). Structured clinical interview for DSM-IV diagnosis (SCID-I).

[CIT0017] Foa E. B, Keane T. M, Friedman M. J, Cohen J. A (2009). Effective treatments for PTSD: Practice guidelines from the International Society for Traumatic Stress Studies.

[CIT0018] Ford J. D, Kidd P (1998). Early childhood trauma and disorders of extreme stress as predictors of treatment outcome with chronic posttraumatic stress disorder. Journal of Traumatic Stress.

[CIT0019] Herman J. L (1992). Complex PTSD: A syndrome in survivors of prolonged and repeated trauma. Journal of Traumatic Stress.

[CIT0020] Hyer L, Davis H, Albrecht W, Boudewyns P, Woods G (1994). Cluster analysis of MCMI and MCMI-II on chronic PTSD victims. Journal of Clinical Psychology.

[CIT0021] Karatzias A, Power K, McGoldrick T, Brown K, Buchanan R, Sharp D (2007). Predicting treatment outcome on three measures for post-traumatic stress disorder. European Archives of Psychiatry and Clinical Neuroscience.

[CIT0022] Kessler R. C, Sonnega A, Bromet E, Hughes M, Nelson C. B (1995). Posttraumatic stress disorder in the National Comorbidity Survey. Archives of General Psychiatry.

[CIT0023] McDonagh A, Friedman M, McHugo G, Ford J, Sengupta A, Mueser K (2005). Randomized trial of cognitive-behavioral therapy for chronic posttraumatic stress disorder in adult female survivors of childhood sexual abuse. Journal of Consulting and Clinical Psychology.

[CIT0024] Miller M. W, Resick P. A (2007). Internalizing and externalizing subtypes in female sexual assault survivors: Implications for the understanding of complex PTSD. Behavior Therapy.

[CIT0025] Olatunji B. O, Cisler J. M, Deacon B. J (2010). Efficacy of cognitive behavioral therapy for anxiety disorders: A review of meta-analytic findings. The Psychiatric Clinics of North America.

[CIT0026] Pelcovitz D, Van der Kolk B, Roth S, Mandel F, Kaplan S, Resick P (1997). Development of a criteria set and a structured interview for disorders of extreme stress (SIDES). Journal of Traumatic Stress.

[CIT0027] Pfohl N, Blum N, Zimmerman M (1997). Structured interview for DSM-IV personality (SIDP-IV).

[CIT0028] Resick P. A, Galovski T. E, O'Brien U. M, Scher C. D, Clum G. A, Young-Xu Y (2008). A randomized clinical trial to dismantle components of cognitive processing therapy for posttraumatic stress disorder in female victims of interpersonal violence. Journal of Consulting and Clinical Psychology.

[CIT0029] Steinberg M, Rounsaville B, Cicchetti D. V (1990). The structured clinical interview for DSM III-R dissociative disorders: Preliminary report on a new diagnostic instrument. American Journal of Psychiatry.

[CIT0030] Talbot N. L, Duberstein P. R, Butzel J. S, Cox C, Giles D. E (2003). Personality traits and symptom reduction in a group treatment for women with histories of childhood sexual abuse. Comprehensive Psychiatry.

[CIT0031] Tarrier N, Pilgrim H, Sommerfield C, Faragher B, Reynolds M, Graham E (1999). A randomized trial of cognitive therapy and imaginal exposure in the treatment of chronic posttraumatic stress disorder. Journal of Consulting and Clinical Psychology.

[CIT0032] Thomaes K, Dorrepaal E, Draijer N, De Ruiter M. B, Elzinga B. M, Sjoerds Z (2013). Increased anterior cingulate cortex and hippocampus activation in complex PTSD during encoding of negative words. Social Cognitive and Affective Neuroscience.

[CIT0033] Thomaes K, Dorrepaal E, Draijer N, De Ruiter M. B, Van Balkom A. J, Smit J. H (2010). Reduced anterior cingulate and orbitofrontal volumes in child abuse-related complex PTSD. Journal of Clinical Psychiatry.

[CIT0034] Van Der Kolk B. A, Roth S, Pelcovitz D, Sunday S, Spinazzola J (2005). Disorders of extreme stress: The empirical foundation of a complex adaptation to trauma. Journal of Traumatic Stress.

[CIT0035] Van Der Kolk B. A, Spinazzola J, Blaustein M. E, Hopper J. W, Hopper E. K, Korn D. L (2007). A randomized clinical trial of eye movement desensitization and reprocessing (EMDR), fluoxetine, and pill placebo in the treatment of posttraumatic stress disorder: Treatment effects and long-term maintenance. Journal of Clinical Psychiatry.

[CIT0036] Van Minnen A, Arntz A, Keijsers G. P (2002). Prolonged exposure in patients with chronic PTSD: Predictors of treatment outcome and dropout. Behavioural Research and Therapy.

[CIT0037] Weertman A, Arntz A, Schouten E, Dreessen L (2005). Influences of beliefs and personality disorders on treatment outcome in anxiety patients. Journal of Consulting and Clinical Psychology.

[CIT0038] Wolfsdorf C.A, Zlotnick C (2001). Affect management in group therapy for women with posttraumatic stress disorder and histories of childhood sexual abuse. Journal of Clinical Psychology.

[CIT0039] Zlotnick C, Simpson E, Begin A, Costello E (1995). Affect-management group treatment for survivors of sexual abuse with posttraumatic stress disorder.

[CIT0040] Zlotnick C, Shea T. M, Rosen K, Simpson E, Mulrenin K, Begin A (1997). An affect-management group for women with posttraumatic stress disorder and histories of childhood sexual abuse. Journal of Traumatic Stress.

